# Genome-Wide Association Study of Genetic Predictors of Anti–Tumor Necrosis Factor Treatment Efficacy in Rheumatoid Arthritis Identifies Associations with Polymorphisms at Seven Loci

**DOI:** 10.1002/art.30130

**Published:** 2011-03

**Authors:** Darren Plant, John Bowes, Catherine Potter, Kimme L Hyrich, Ann W Morgan, Anthony G Wilson, John D Isaacs, Anne Barton, Wellcome Trust Case Control Consortium, the British Society for Rheumatology Biologics Register

**Affiliations:** 1 University of ManchesterManchester, UK; 2 Newcastle UniversityNewcastle-upon-Tyne, UK; 3 University of LeedsLeeds, UK; 4 University of SheffieldSheffield, UK

## Abstract

**Objective:**

Anti–tumor necrosis factor (anti-TNF) agents are successful therapies in rheumatoid arthritis (RA); however, inadequate response occurs in 30–40% of patients treated. Knowledge of the genetic factors that influence response may facilitate personalized therapy. The purpose of this study was to identify genetic predictors of response to anti-TNF therapy in RA and to validate our findings in independent cohorts.

**Methods:**

Data from genome-wide association (GWA) studies were available from the Wellcome Trust Case Control Consortium for 566 anti-TNF–treated RA patients. Multivariate linear regression analysis of changes in the Disease Activity Score in 28 joints at 6 months was conducted at each single-nucleotide polymorphism (SNP) using an additive model. Associated markers (*P* < 10^−3^) were genotyped in 2 independent replication cohorts (n = 379 and n = 341), and a combined analysis was performed.

**Results:**

Of 171 successfully genotyped markers demonstrating association with treatment response in the GWA data, 7 were corroborated in the combined analysis. The strongest effect was at rs17301249, mapping to the *EYA4* gene on chromosome 6: the minor allele conferred improved response to treatment (coefficient −0.27, *P* = 5.67^−05^). The minor allele of rs1532269, mapping to the *PDZD2* gene, was associated with a reduced treatment response (coefficient 0.20, *P* = 7.37^−04^). The remaining associated SNPs mapped to intergenic regions on chromosomes 1, 4, 11, and 12.

**Conclusion:**

Using a genome-wide strategy, we have identified and validated the association of 7 genetic loci with response to anti-TNF treatment in RA. Additional confirmation of these findings in further cohorts will be required.

Rheumatoid arthritis (RA) is a chronic, autoimmune, inflammatory disease of the synovial joints, affecting ∼1% of the Caucasian population (1). Disease progression leads to joint destruction, resulting in functional disability, and RA is a significant cause of comorbidity and mortality.

The mainstay of treatment for RA is disease-modifying antirheumatic drugs (DMARDs), such as methotrexate, which aim to control the widespread inflammation characteristic of the disease. However, such drugs are often associated with significant side effects and are not universally effective. In recent years, the anti–tumor necrosis factor (TNF) biologic drugs, including etanercept, infliximab, and adalimumab, have proven highly successful in potently suppressing both inflammation and joint damage, with 60–70% of patients responding to these therapies (2,3). However, this means that a substantial proportion of patients show no response or only a limited response, and given their expense (approximately £10,000 per patient per year in the UK; similar in other countries), the identification of predictors of response at baseline could be of great clinical and economic benefit by allowing the targeting of these therapies to the patients who are most likely to respond.

Clinical predictors of response, such as concurrent methotrexate or nonsteroidal antiinflammatory drug therapy, functional disability, and smoking habits, account for only a small proportion of the variance in treatment response (4,5). Additional factors, such as genetic and serologic markers, are also likely to influence response.

Most studies of genetic predictors of anti-TNF response performed to date have focused on candidate genes known to play or thought to play a role in susceptibility to RA, such as the *HLA–DRB1* shared epitope in the HLA region (6). Other studies have investigated the *TNF* gene itself (7,8), additional genes in the TNF signaling pathway (9), and other cytokines (10). Despite the many studies that have been conducted, no gene that influences anti-TNF response in RA has been definitively identified and replicated, although evidence for a role of the *TNF* −308 polymorphism is compelling (5). Small sample sizes and a focus on few variants in a narrow selection of candidate genes are the most likely explanations for this limited success, which highlights the need for a new strategy.

Over the last 2–3 years, genome-wide association (GWA) studies have been highly successful in identifying susceptibility genes in complex diseases. Such studies aim to interrogate thousands of genetic markers covering the majority of the whole genome to assess their relationship to the particular outcome of interest. Thus, GWA studies take an unbiased view of the whole genome and therefore have a higher probability of detecting an association with a genetic marker, providing that the studies are sufficiently powered.

In 2007, the Wellcome Trust Case Control Consortium (WTCCC) in the UK published the results of a large collaborative effort aimed at identifying common susceptibility polymorphisms implicated in 7 complex diseases (11). A case–control GWA study of ∼500,000 single-nucleotide polymorphisms (SNPs) was performed in 3,000 control subjects and in 2,000 patients in each disease group, one of which was RA. Of the 2,000 RA cases contributed by our group, 566 were patients receiving anti-TNF treatment and had available data regarding treatment response.

The aims of the current study, therefore, were first, to identify candidate genetic predictors of response to anti-TNF therapy from the available GWA data and second, to validate these findings using independent cohorts of anti-TNF–treated RA patients.

## PATIENTS AND METHODS

### Study design

This study used a 3-stage design. In the first stage, GWA analysis of change in the Disease Activity Score in 28 joints (DAS28) (12) between baseline and 6 months was undertaken in 566 anti-TNF–treated RA patients who where included as part of the WTCCC study (stage 1 cohort). In the second stage, markers demonstrating evidence of association (*P* < 10^−3^) in stage 1 were genotyped in an independent cohort of 410 individuals (stage 2 cohort), and a meta-analysis of the 2 datasets was performed. In stage 3, markers for which the association signal was strengthened by meta-analysis, as compared to the association signal observed in the stage 1 cohort alone, were investigated in a third cohort of 364 anti-TNF–treated RA patients (stage 3 cohort), and a second meta-analysis of all 3 datasets was performed. The choice of a 3-stage design was pragmatic, since the study is actively recruiting, and further genotyping/analysis was undertaken as sufficient additional samples became available for inclusion.

### Patients

The British Society for Rheumatology Biologics Register (BSRBR) was initiated with the aim of assessing the adverse events associated with treatment with the 3 anti-TNF biologic agents. The BSRBR has detailed clinical and response criteria on 4,000 patients with RA receiving each drug. Collaborations were established with a subset of the larger prescribing centers as part of the Biologics in Rheumatoid Arthritis Genetics and Genomics Study Syndicate (BRAGGSS; see Appendix A for the BRAGGSS investigators and study centers). Blood samples for DNA extraction were obtained from anti-TNF–treated RA patients who met the following 3 inclusion criteria: 1) RA confirmed by a physician, 2) currently receiving or about to begin receiving treatment with 1 of 3 anti-TNF drugs (etanercept, infliximab, or adalimumab), and 3) Caucasian origin, thus reducing the potential for spurious associations arising as a result of population stratification. Patients were ineligible for this study if they had stopped treatment during the first 6 months for reasons other than lack of efficacy.

### Marker selection and genotyping

Genotyping of DNA samples included in the original GWA study of RA was described in detail in the online methods for the WTCCC report (11). Briefly, 250 ng of DNA was genotyped using an Affymetrix GeneChip 500K Mapping Array Set, which interrogates 500,000 SNPs. After quality control measures, a total of 459,446 SNPs remained available for analysis.

Markers demonstrating a significant association (*P* < 10^−3^) with treatment response in the initial GWA stage of the study were selected for replication. Markers with minor allele frequencies of <10% were excluded from further investigation, as were markers with genotype frequencies that deviated from Hardy-Weinberg equilibrium at *P* < 10^−3^.

To reduce the genotyping burden at the second stage of the study, linkage disequilibrium between association signals from the initial stage was analyzed, and tag SNPs demonstrating correlation (r^2^ > 0.8) with the remaining associated SNPs were selected for genotyping. These analyses were performed using Plink statistical software, release v1.07 (online at http://pngu.mgh.harvard.edu/purcell/plink/).

DNA samples from the patients evaluated in stages 2 and 3 were genotyped using a Sequenom MassArray iPlex system. In each reaction, 10 ng of DNA was used, and the protocol was followed according to the manufacturer's instructions (http://www.Sequenom.com).

Quality control measures were used at the genotyping stages of the study. Samples and SNPs with a genotyping success rate of < 80% were excluded from analyses.

### Statistical analysis

Multivariate linear regression analysis was used to assess the effect of each SNP genotype on response to treatment, using the absolute change in the DAS28 at 6 months of followup (a continuous variable) as the outcome measure. Regression analyses were adjusted for covariates previously identified as being independent predictors of change in the DAS28: baseline DAS28, Health Assessment Questionnaire (HAQ) score (13), and concurrent DMARD therapy. These analyses were performed using Plink statistical software, release v1.07. In the stage 1 cohort, sex and smoking history were not significantly associated with change in the DAS28, and serology data (e.g., anti–cyclic citrullinated peptide antibody and rheumatoid factor) was available for <85% of patients; therefore, these data were not included as covariates.

To establish a drug type–specific effect at any of the associated loci, data for all samples were combined, and an interaction term was fitted between SNP loci and drug type (i.e., etanercept, infliximab, or adalimumab). These analyses were performed using Stata statistical software, release 9, 2005 (StataCorp; online at http://www.stata.com)

Power calculations were performed using Quanto version 1.2.3 (online at http://hydra.usc.edu/gxe) under an additive model for a range of marker allele frequencies.

## RESULTS

For stage 1 of the study, the initial GWA, DNA samples from 566 RA patients receiving anti-TNF treatment were available for study. The initial analysis had >90% power to detect a difference of ≥0.6 units in the absolute change in the DAS28 at the significance threshold (*P* < 10^−3^) for allele frequencies ≥15%.

For investigation of association signals arising from the initial GWA, 410 patients who were also treated with one of the anti-TNF biologic agents were genotyped. Genotyping quality control failed in 7 patients, leaving 403 patients, of which 379 had complete information for analysis. Finally, 364 patients were genotyped in stage 3 of the study. Genotyping quality control failed in 16 of them, and of the remaining 348 patients, 341 had sufficient data on baseline covariates and on treatment response for analysis. Patient characteristics for the 3 cohorts available for analysis are given in Table 1.

**Table 1:** Baseline characteristics of the patients in each cohort

	Initial cohort				Stage 2 cohort				Stage 3 cohort			
			
Baseline characteristic*	Etanercept	Infliximab	Adalimumab	Combined	Etanercept	Infliximab	Adalimumab	Combined	Etanercept	Infliximab	Adalimumab	Combined
No. (%) of cases	244 (43)	260 (46)	62 (11)	566	161 (43)	149 (39)	69 (18)	379	84 (25)	92 (27)	165 (48)	341
Age, mean ± SD years	57 ± 11	57 ± 11	59 ± 12	58 ± 11	55 ± 11	54 ± 12	57 ± 12	55 ± 11	57 ± 10	56 ± 11	57 ± 10	57 ± 11
No. (%) female	192 (79)	199 (77)	48 (77)	439 (78)	119 (74)	114 (77)	50 (72)	283 (75)	64 (76)	73 (79)	131 (79)	268 (79)
Smoking status, no. (%) of patients												
Current smokers	45 (19)	45 (17)	7 (12)	97 (17)	20 (12)	27 (18)	10 (15)	57 (15)	14 (17)	10 (11)	26 (16)	50 (15)
Ever smoked	139 (56)	147 (57)	33 (55)	319 (57)	102 (63)	82 (55)	42 (62)	226 (60)	47 (56)	49 (53)	100 (62)	196 (58)
Disease duration, mean ± SD years	13 ± 9	15 ± 10	12 ± 10	14 ± 10	15 ± 9	13 ± 10	15 ± 13	14 ± 10	13 ± 9	14 ± 11	12 ± 10	13 ± 10
Health status scores, mean ± SD												
DAS28	6.7 ± 0.9	6.7 ± 0.9	6.5 ± 1	6.7 ± 0.9	6.7 ± 1	6.8 ± 1	6.6 ± 0.9	6.7 ± 1	6.8 ± 0.9	6.6 ± 1	9.4 ± 0.9	6.6 ± 1
HAQ	2.0 ± 0.6	2.1 ± 0.5	2.1 ± 0.5	2.1 ± 0.6	2.0 ± 0.5	2.0 ± 0.6	2.0 ± 0.6	2.0 ± 0.5	2.1 ± 0.6	1.9 ± 0.5	1.8 ± 0.6	1.9 ± 0.6
Medications, no. (%) of patients												
Concurrent DMARD(s)	127 (52)	242 (93)	40 (65)	409 (72)	72 (45)	138 (93)	47 (68)	257 (68)	49 (58)	88 (96)	127 (77)	264 (77)
Concurrent steroids	96 (39)	119 (46)	25 (40)	240 (42)	62 (39)	61 (41)	31 (45)	154 (41)	37 (44)	41 (45)	59 (36)	137 (40)
Previous biologic agent	13 (5)	10 (4)	3 (4)	26 (5)	8 (5)	5 (3)	10 (14)	23 (6)	3 (4)	12 (13)	9 (6)	24 (7)

* DAS28 = Disease Activity Score in 28 joints; HAQ = Health Assessment Questionnaire; DMARD = disease-modifying antirheumatic drug.

### Stage 1, initial GWA analysis

Of the 459,446 SNP markers analyzed, 382 SNPs demonstrated significant association (*P* < 10^−3^) with response to treatment, as measured by the absolute change in the DAS28 at 6 months. After excluding SNPs with a minor allele frequency of <0.10 and those deviating from Hardy-Weinberg equilibrium (*P* < 10^−3^), 247 SNPs remained for further investigation. A list of these SNPs and their corresponding *P* values for the additive model of association are shown in Supplementary Table 1 (available on the *Arthritis & Rheumatism* web site at http://onlinelibrary.wiley.com/journal/10.1002/(ISSN)1529-0131).

Using a tagging strategy based on linkage disequilibrium between associated SNPs (R^2^ ≥ 0.8), the number of SNPs prioritized for genotyping in the first replication cohort was reduced from 247 to 183 (Supplementary Table 1).

### Stage 2, first meta-analysis

Of the 183 SNP markers identified in stage 1, 12 (i.e., rs11254686, rs11170826, rs10512080, rs10167992, rs9471419, rs6838850, rs6432150, rs4957798, rs3911167, rs2810034, rs16892786, and rs12312888) failed to genotype in the stage 2 cohort, leaving a total of 171 SNPs for meta-analysis (Supplementary Table 1).

Ten SNP markers (i.e., rs7305646, rs1350948, rs7962316, rs17301249, rs1532269, rs4694890, rs7070180, rs12081765, rs1024125, and rs10739625) demonstrated an improved association signal with the response to anti-TNF treatment in the meta-analysis of the discovery and stage 2 cohorts over and above that observed in the discovery cohort alone (*P* < 0.001) under an additive model of association (Table 2).

**Table 2:** Genotype and response to treatment association data in the initial and stage 2 cohort for 10 associated SNPs*

				Initial cohort (n = 566)					Stage 2 cohort (n = 379)						
							
							Additive model					Additive model		Meta-analysis, fixed effects	
												
SNP	Chr.	Position, bp	Genotype	Count	Baseline DAS28, mean ± SD	Change in DAS28, mean ± SD	*P*	Coefficient (95% CI)	Count	Baseline DAS28, mean ± SD	Change in DAS28, mean ± SD	*P*	Coefficient (95% CI)	*P*	Coefficient
rs12081765	1	162,073,507	11	182	6.6 ± 0.9	−2.7 ± 1.6			109	6.8 ± 1.0	−2.7 ± 1.3				
			12	283	6.8 ± 1.0	−2.5 ± 1.5	7.52^−04^	0.29 (0.12, 0.46)	191	6.7 ± 1.0	−2.4 ± 1.5	6.17^−02^	0.19 (−0.01, 0.39)	1.47^−04^	0.25
			22	101	6.7 ± 0.9	−2.1 ± 1.5			77	6.8 ± 1.0	−2.4 ± 1.6				
rs1532269	5	32,054,598	11	217	6.7 ± 0.9	−2.7 ± 1.5			158	6.7 ± 1.1	−2.6 ± 1.5				
			12	278	6.7 ± 0.9	−2.5 ± 1.4	7.11^−04^	0.30 (0.13, 0.48)	178	6.8 ± 0.9	−2.5 ± 1.5	7.86^−02^	0.19 (−0.02, 0.39)	1.85^−04^	0.26
			22	70	6.5 ± 0.9	−2.0 ± 1.7			42	6.7 ± 1.0	−2.14 ± 1.4				
rs17301249	6	133,654,607	11	364	6.7 ± 0.9	−2.3 ± 1.5			249	6.7 ± 1.0	−2.4 ± 1.5				
			12	179	6.7 ± 0.9	−2.8 ± 1.5	3.37^−04^	−0.38 (−0.58, −0.17)	112	6.7 ± 0.9	−2.6 ± 1.6	4.51^−02^	−0.24 (−0.48, −0.01)	5.17^−05^	−0.32
			22	23	6.7 ± 1.0	−3.0 ± 1.3			17	7.0 ± 1.0	−3.0 ± 1.0				
rs7305646†	12	17,155,604	11	159	6.7 ± 0.9	−2.3 ± 1.6			90	6.9 ± 1.1	−2.3 ± 1.6				
			12	275	6.8 ± 0.9	−2.5 ± 1.5	9.16^−04^	−0.28 (−0.44, −0.11)	201	6.7 ± 0.9	−2.5 ± 1.5	4.91^−02^	−0.21 (−0.41, −0.001)	1.25^−04^	−0.25
			22	128	6.6 ± 1.0	−2.8 ± 1.5			83	6.6 ± 1.1	−2.5 ± 1.5				
rs4694890	4	48,067,195	11	150	6.6 ± 0.9	−2.2 ± 1.5			103	6.7 ± 1.0	−2.3 ± 1.4				
			12	301	6.7 ± 0.9	−2.5 ± 1.5	7.09^−04^	−0.30 (−0.47, −0.13)	187	6.7 ± 1.0	−2.4 ± 1.5	4.36^−02^	−0.20 (−0.39, −0.01)	9.95^−05^	−0.25
			22	115	6.9 ± 1.0	−2.9 ± 1.4			89	6.7 ± 1.0	−2.8 ± 1.5				
rs1350948	11	23,474,981	11	400	6.7 ± 1.0	−2.6 ± 1.5			277	6.7 ± 1.0	−2.5 ± 1.5				
			12	128	6.8 ± 0.9	−2.2 ± 1.6	8.65^−04^	0.37 (0.15, 0.59)	89	6.9 ± 0.9	−2.3 ± 1.4	4.08^−02^	0.27 (0.01, 0.53)	1.01^−04^	0.33
			22	22	6.6 ± 1.0	−1.7 ± 1.6			13	6.5 ± 1.1	−2.1 ± 0.9				
rs7962316	12	90,922,423	11	230	6.7 ± 0.9	−2.7 ± 1.6			178	6.6 ± 1.1	−2.5 ± 1.5				
			12	257	6.7 ± 0.9	−2.4 ± 1.5	5.09^−04^	0.30 (0.13, 0.47)	145	6.9 ± 0.9	−2.5 ± 1.4	8.60^−02^	0.17 (−0.02, 0.37)	1.64^−04^	0.25
			22	77	6.6 ± 1.0	−2.2 ± 1.5			53	6.7 ± 1.0	−2.2 ± 1.6				
rs7070180	10	117,842,460	11	319	6.6 ± 0.9	−2.7 ± 1.4			199	6.8 ± 1.0	−2.6 ± 1.4				
			12	209	6.7 ± 1.0	−2.3 ± 1.7	2.24^−04^	0.35 (0.17, 0.54)	161	6.6 ± 1.0	−2.4 ± 1.6	8.24^−02^	0.21 (−0.03, 0.45)	6.42^−05^	0.30
			22	38	7.1 ± 1.1	−2.3 ± 1.3			16	7.1 ± 1.0	−2.2 ± 1.4				
rs1024125	2	174,533,706	11	271	6.7 ± 1.0	−2.3 ± 1.5			187	6.8 ± 1.0	−2.4 ± 1.5				
			12	228	6.7 ± 0.9	−2.6 ± 1.5	8.54^−04^	−30 (−0.47, −0.12)	160	6.7 ± 1.0	−2.5 ± 1.5	1.60^−01^	−0.15 (0.11, −0.37)	4.81^−04^	−0.24
			22	61	6.8 ± 1.0	−2.8 ± 1.7			32	6.6 ± 1.1	−2.7 ± 1.4				
rs10739625	9	123,153,234	11	413	6.7 ± 0.9	−2.6 ± 1.5			269	6.7 ± 1.0	−2.6 ± 1.5				
			12	135	6.7 ± 0.9	−2.3 ± 1.6	9.92^−04^	0.38 (0.15, 0.60)	99	6.7 ± 1.0	−2.2 ± 1.5	9.23^−02^	0.23 (0.13, −0.04)	2.96^−04^	0.31
			22	18	7.1 ± 0.9	−2.1 ± 1.6			10	7.0 ± 1.2	−2.7 ± 1.4				

* Chr. = chromosome; DAS28 = Disease Activity Score in 28 joints; 95% CI = 95% confidence interval. 1 = major allele, 2 = minor allele.

† The genotyping assay for this single-nucleotide polymorphism (SNP) failed in the first replication cohort; therefore, a perfect proxy (rs4522221; r^2^ = 1) was genotyped.

### Stage 3, second meta-analysis

To further investigate the observed associations with treatment response, the 10 SNPs were genotyped in a second independent cohort of anti-TNF–treated RA patients. Three SNPs (rs7070180, rs1024125, and rs10739625) failed to genotype, leaving 7 SNPs for analysis. A second meta-analysis of these data along with the discovery and stage 2 cohort data for the 7 SNPs was performed. The association signal for each marker (i.e., rs12081765, rs1350948, rs1532269, rs17301249, rs4694890, rs7305646, and rs7962316) remained the same or diminished in significance in the second meta-analysis, as compared with that observed in the first meta-analysis (Table 3). For the SNP markers rs4694890, rs1350948, and rs7962316, the effect was observed in the opposite direction in the stage 3 cohort, as compared with the initial and stage 2 cohorts, suggesting that these associations may be spurious.

**3:** Genotype and response to treatment association data in the stage 3 cohort for 7 associated SNPs*

				Stage 3 cohort (n = 341)						
					
							Additive model		Second meta-analysis, fixed-effects	
								
SNP	Chr.	Position, bp	Genotype	Count	Baseline DAS28, mean ± SD	Change in DAS28, mean ± SD	*P*	Coefficient (95% CI)	*P*	Coefficient
rs12081765	1	162,073,507	11	95	6.5 ± 0.8	−2.6 ± 1.5				
			12	155	6.6 ± 1.1	−2.5 ± 1.4	7.12^−01^	0.04 (−0.16, 0.23)	7.39^−04^	0.18
			22	77	6.5 ± 0.9	−2.5 ± 1.4				
rs1532269	5	32,054,598	11	142	6.4 ± 1.0	−2.5 ± 1.4				
			12	150	6.7 ± 0.9	−2.5 ± 1.4	7.01^−01^	0.04 (−0.17, 0.26)	7.37^−04^	0.20
			22	35	6.6 ± 1.1	−2.6 ± 1.4				
rs17301249	6	133,654,607	11	219	6.5 ± 1.0	−2.5 ± 1.4				
			12	97	6.5 ± 1.0	−2.8 ± 1.4	2.61^−01^	−0.14 (−0.39, 0.11)	5.67^−05^	−0.27
			22	14	7.0 ± 0.9	−2.5 ± 1.5				
rs7305646†	12	17,155,604	11	83	6.7 ± 1.0	−2.5 ± 1.2				
			12	162	6.5 ± 1.0	−2.4 ± 1.4	2.92^−01^	−0.11 (−0.31, 0.09)	1.47^−04^	−0.21
			22	82	6.5 ± 0.8	−2.7 ± 1.6				
rs4694890	4	48,067,195	11	102	6.5 ± 1.0	−2.5 ± 1.5				
			12	153	6.5 ± 1.0	−2.7 ± 1.3	3.45^−01^	0.09 (−0.10, 0.29)	6.47^−03^	−0.15
			22	74	6.7 ± 0.9	−2.4 ± 1.4				
rs1350948	11	23,474,981	11	219	6.5 ± 1.0	−2.5 ± 1.4				
			12	103	6.6 ± 1.0	−2.6 ± 1.4	2.29^−01^	−0.16 (−0.42, 0.10)	8.64^−03^	0.19
			22	9	6.6 ± 0.7	−3.1 ± 1.4				
rs7962316	12	90,922,423	11	146	6.5 ± 1.0	−2.5 ± 1.4				
			12	124	6.5 ± 0.9	−2.5 ± 1.4	1.51^−01^	−0.14 (−0.33, 0.05)	2.05^−02^	0.13
			22	57	6.6 ± 1.0	−2.8 ± 1.3				

* Chr. = chromosome; DAS28 = Disease Activity Score in 28 joints; 95% CI = 95% confidence interval. 1 = major allele, 2 = minor allele.

† The genotyping assay for this single-nucleotide polymorphism (SNP) failed in both replication cohorts; therefore, 2 perfect proxies (rs4522221; r^2^ = 1 [first replication cohort] and rs7133213; r^2^ = 1 [second replication cohort]) were genotyped.

### Clinical significance

Taking into account the clinical and demographic factors previously shown by our group and others to influence response to anti-TNF treatment in RA patients (DAS28 score at baseline, HAQ score, sex, concurrent DMARD therapy, rheumatoid factor positivity, and smoking habits), the variance in the absolute change in the DAS28 at 6 months of followup in the combined cohort was 15%. Incorporating into the model the 7 genetic loci identified by the current study increased the variance explained to 20%. When the model was restricted to include only those SNPs with between-study continuity in the direction of effect (i.e., rs12081765, rs1532269, rs17301249, and rs7305646), the variance in response to treatment was 19%.

Each treatment response–associated SNP was investigated for drug type–specific effects by fitting an interactive term in the analysis. No statistical correlation between the type of therapy used and the SNP marker was observed (data not shown).

## DISCUSSION

We performed a multistage comprehensive GWA study of response to anti-TNF treatment in patients with RA. In combining the results of the initial GWA study and 2 independent cohorts, we demonstrated evidence of association at 7 genetic loci not previously implicated in response to these drugs, with the significance of the association increased for markers rs12081765, rs17301249, and rs7305646 in the second meta-analysis, as compared to the initial GWA results (Tables 2 and 3).

Two of the SNP markers map within genes: the PDZ domain–containing protein 2 (*PDZD2*) and eyes absent homolog 4 (*EYA4*). The SNP marker rs1532269, which is associated with a reduced response to TNF blockade, is an intronic polymorphism mapping to the *PDZD2* gene. The SNP resides in a region of linkage disequilibrium confined to the latter portion of *PDZD2* and demonstrates correlation (r^2^ > 0.8) with 5 other intronic SNPs within *PDZD2*. The *PDZD2* gene has been reported to influence the secretion of insulin in an animal model in which pancreatic islet cells of *Pdzd2*-deficient mice produce more insulin than do normal islets and display increased insulin secretion at low concentrations of glucose (14). Insulin resistance and elevated insulin levels are features of severe disease in early RA, if untreated, and are driven primarily by systemic inflammation (15). In RA, dramatic reductions in serum insulin levels are observed following anti-TNF treatment (16). Therefore, a potentially interesting connection exists between insulin levels and disease severity in patients with RA, and this connection may have a genetic basis.

The *EYA4* SNP associated with an improved treatment response in the current study, rs17301249, is an intronic variant tagging an additional intronic *EYA4* polymorphism (rs9375955) that was associated with response to treatment at the initial GWA analysis stage. In the HapMap CEU (Utah residents with ancestry from northern and western Europe) data set (release 22), the two SNPs are strongly correlated with other intronic *EYA4* SNPs and SNPs upstream of the transcription start site; the region of high linkage disequilibrium does not stretch to any nearby genes. Mapping to chromosome 6q23.2, *EYA4* was originally discovered as a co–transcription factor and is observed to stimulate the expression of interferon-β (IFNβ) and CXCL10 in response to undigested DNA of apoptotic cells (17). Once engulfed by macrophages, the DNA molecule from dead cells is digested by the activity of DNase II. Mice that are deficient in DNase II are prone to developing chronic arthritis as a result of the production of TNF and IFNβ (17). Cross-talk between type I IFN and TNF has recently been investigated in patients with RA, in whom elevated expression of type I IFN response genes has been shown to correlate with a poor clinical response following TNF blockade (18). The observed genetic association in the current study could therefore indicate a link between DNA-induced innate immune responses and the efficacy of TNF blockade.

Three SNPs (i.e., rs4694890, rs1350948, and rs7962316) showed an improved association signal in the first meta-analysis, over and above that seen in the initial GWA data; however, these SNPs failed to associate with treatment response in the stage 3 cohort (n = 341), and with the effect observed in the direction opposite that expected (Tables 2 and 3). The between-cohort differences in association signals for these loci are likely to be due to the small sample sizes, and while interpretation of these data should be treated cautiously, they suggest that these may be false-positive signals.

Interpreting the validated association signals located in intergenic loci on chromosomes 1 (rs12081765), 4 (rs4694890), 11 (rs1350948), and 12 (rs7962316 and rs7305646) is challenging, since they do not map close to obvious candidate genes. Nonetheless, we have found evidence of association with anti-TNF response, and it is possible to speculate that they represent long-range regulatory elements for other genes that they may lie more closely to when the 3-dimensional conformation rather than the linear DNA sequence is taken into account. Investigation of chromosomal conformation will be required to explore this further.

Our study has some limitations that require discussion. First, in order to avoid false-negative associations at the initial GWA stage, a relatively lenient statistical threshold of *P* < 10^−3^ was chosen, thus running the risk of generating a high proportion of false-positive associations. Our strategy was to validate association signals rather than imposing a strict level of significance at the first stage. In the combined analysis of all 3 cohorts by meta-analysis, evidence of association was increased over that obtained in the initial cohort alone for 3 of the 7 markers investigated (rs12081765, rs17301249, and rs7305646). Therefore, it is important that other researchers in the field also aim to provide additional verification of our findings in independent collections of anti-TNF–treated RA patients.

Second, although a powerful means of measuring treatment response, the DAS28 score has the limitation that it is a composite score, relying on information about swollen and tender joint counts, patient-reported general health status, and the erythrocyte sedimentation rate (ESR). Hence, it should not be surprising that genetic effects influencing this complex phenotype are likely to be individually modest, as has been found in most studies of disease susceptibility. Where large effect sizes have been observed in pharmacogenetic studies, there is usually a well-defined phenotype (such as a rare adverse event) or detection of an association with a biologic marker, such as the influence of genetic variation in warfarin therapy, as assessed by measurements of the international normalized ratio. In assessing response to anti-TNF therapies, therefore, stronger effect sizes may be seen with individual components of the DAS28 score. For example, the ESR is a biologic marker of inflammation and is therefore an objective measure that may provide a more accurate means of assessing treatment response. Future studies will examine genetic predictors of response using the ESR as the outcome measure to investigate whether that approach highlights the same genetic loci or whether the proportion of signals from the initial phase that are subsequently validated is higher.

To date, our study, which included 1,285 patients, is the largest investigation of genetic predictors of anti-TNF response in RA patients. The sample size used at the initial phase of the study afforded >90% power to detect a change in the DAS28 of 0.6 units, representing a clinically relevant change in disease activity, at allele frequencies ≥0.15. It is possible that effects conferred by less frequent alleles were overlooked.

Despite the limitations, 7 loci have been identified, which, when added into predictive models, substantially increase the variance accounted for over and above clinical variables alone. The next step will be to further explore the 7 loci using a fine-mapping strategy to identify the polymorphism responsible for the effect on treatment response. This will be particularly challenging at the intergenic loci identified, since the associated SNP is often >100 kb from the nearest known gene, or there are no obvious candidate genes in the region. It is therefore likely that functional variants with long-range effects on gene regulation are responsible for the observed associations at these intergenic loci.

In summary, we have performed a multistage association study of response to anti-TNF treatment in RA patients and have identified 7 genetic loci that influence treatment response in our data. As with all studies of genetic determinants of complex phenotypes, our findings require validation in independent cohorts.

## AUTHOR CONTRIBUTIONS

All authors were involved in drafting the article or revising it critically for important intellectual content, and all authors approved the final version to be published. Dr. Barton had full access to all of the data in the study and takes responsibility for the integrity of the data and the accuracy of the data analysis.

**Study conception and design.**Plant, Morgan, Wilson, Isaacs, Barton.

**Acquisition of data.**Plant, Bowes, Potter, Hyrich, Wilson, Barton.

**Analysis and interpretation of data.**Plant, Bowes, Hyrich, Morgan, Isaacs, Barton.

## APPENDIX A: THE BIOLOGICS IN RHEUMATOID ARTHRITIS GENETICS AND GENOMICS STUDY SYNDICATE

Members of the BRAGGSS are as follows: from the Cambridge University Hospitals NHS Foundation Trust, Dr. A. J. Crisp, Prof. J. S. H. Gaston, Dr. F. C. Hall, Dr. B. L. Hazleman, Dr. J. R. Jenner, Dr. A. Ostor, Dr. B. Silverman, and Dr. C. Speed; from the County Durham and Darlington Acute Hospitals NHS Trust, Dr. D. Armstrong, Dr. A. J. Chuck, and Dr. S. Hailwood; from the Derby Hospitals NHS Foundation Trust, Dr. L. J. Badcock, Dr. C. M. Deighton, Dr. S. C. O'Reilly, Dr. M. R. Regan, Dr. Snaith, Dr. G. D. Summers, and Dr. R. A. Williams; from the Doncaster and Bassetlaw Hospitals NHS Foundation Trust, Dr. J. R. Lambert; from the Gateshead Health NHS Trust, Dr. J. Hamilton, Dr. C. R. Heycock, Dr. C. A. Kelly, and Dr. V. Saravanan; from the Hereford Hospitals NHS Trust, Dr. D. H. Rees and Dr. R. B. Williams; from the Mid Staffordshire General Hospitals NHS Trust, Dr. S. V. Chalam, Dr. D. Mulherin, Dr. T. Price, and Dr. T. Sheeran; from the Morecambe Bay Hospitals NHS Trust, Dr. M. Bukhari, Dr. W. N. Dodds, and Dr. J. P. Halsey; from the Norfolk and Norwich University Hospital NHS Trust, Dr. K. Gaffney, Prof. A. J. Macgregor, Dr. T. Marshall, Dr. P. Merry, and Prof. D. G. I. Scott; from the Pennine Acute Hospitals NHS Trust, Dr. B. Harrison, Dr. M. Pattrick, and Dr. H. N. Snowden; from the Peterborough and Stamford Hospitals NHS Foundation Trust, Dr. N. J. Sheehan and Dr. N. E. Williams; from the Portsmouth Hospitals NHS Trust, Dr. R. G. Hull, Dr. J. M. Ledingham, Dr. F. McCrae, Dr. M. R. Shaban, and Dr. A. L. Thomas; from the Sandwell and West Birmingham Hospitals NHS Trust, Prof. C. D. Buckley, Dr. D. C. Carruthers, Dr. R. Elamanchi, Dr. P. C. Gordon, Dr. K. A. Grindulis, Dr. F. Khattak, Dr. K. Raza, and Dr. D. Situnayake; from the Sheffield Teaching Hospitals NHS Trust, Dr. M. Akil, Dr. R. Amos, Dr. D. E. Bax, Dr. S. Till, Dr. G. Wilson, and Dr. J. Winfield; from the South Tees Hospitals NHS Trust, Dr. F. Clarke, Dr. J. N. Fordham, Dr. M. J. Plant, and Dr. Tuck; from the St. Helens and Knowsley Hospitals NHS Trust, Dr. V. E. Abernethy, Dr. J. K. Dawson, and Dr. M. Lynch; from the Leeds Teaching Hospitals NHS Trust, Dr. S. Bingham, Prof. P. Emery, and Dr. A. Morgan; from the Newcastle upon Tyne Hospitals NHS Trust, Dr. F. Birrell, Mr. P. Crook, Dr. H. E. Foster, Dr. B. Griffiths, Dr. I. D. Griffiths, Dr. M. L. Grove, Prof. J. D. Isaacs, Dr. L. Kay, Dr. A. Myers, Dr. P. N. Platt, and Dr. D. J. Walker; from the University Hospital Birmingham NHS Foundation Trust, Dr. Bowman, Prof. C. D. Buckley, Dr. P. Jobanputra, Dr. R. W. Jubb, and Dr. E. C. Rankin; from the University Hospital of North Staffordshire NHS Trust, Dr. E. H. Carpenter, Dr. P. T. Dawes, Dr. A. Hassell, Prof. E. M. Hay, Dr. S. Kamath, Dr. J. Packham, and Dr. M. F. Shadforth; and from the Whipps Cross University Hospital NHS Trust, Dr. S. P. Donnelly, Dr. D. Doyle, Dr. A. Hakim, and Dr. J. G. Lanham.
